# Co-prescribing of opioids and benzodiazepines/Z-drugs associated with all-cause mortality—A population-based longitudinal study in primary care with weak opioids most commonly prescribed

**DOI:** 10.3389/fphar.2022.932380

**Published:** 2022-09-06

**Authors:** Kristjan Linnet, Heidrun Sjofn Thorsteinsdottir, Johann Agust Sigurdsson, Emil Larus Sigurdsson, Larus Steinthor Gudmundsson

**Affiliations:** ^1^ Development Centre for Primary Healthcare in Iceland, Primary Health Care of the Capital Area, Reykjavik, Iceland; ^2^ Faculty of Pharmaceutical Sciences, School of Health Sciences, University of Iceland, Reykjavik, Iceland; ^3^ General Practice Research Unit, Department of Public Health and Nursing, Norwegian University of Science and Technology (NTNU), Trondheim, Norway; ^4^ Department of Family Medicine, Faculty of Medicine, School of Health Sciences, University of Iceland, Reykjavik, Iceland

**Keywords:** weak opioids, benzodiazepines/Z-drugs, mortality risk, long-term co-prescribing, primary care

## Abstract

**Introduction:** The risk of mortality associated with the co-prescribing of benzodiazepines and opioids has been explored in a number of papers mainly focusing on strong opioids. The mortality risk associated with the use of weak opioids has not been dealt with to a similar extent.

**Objective:** To assess the mortality risk in primary care patients with consistent 3-year co-prescribing of benzodiazepine/Z-drugs (benzodiazepine receptor modulators) and mainly weak opioids (codeine, tramadol).

**Methods:** Of 221,804 patients contacting the primary healthcare centres, 124,436 were selected for further analysis, 88,832 participants fulfilled the inclusion criteria, aged 10–69 years and were divided into four groups with neither any use of benzodiazepines/Z-drugs nor opioids as Group 1, 3 years’ use of opioids and no/minimal benzodiazepines/Z-drugs as Group 2, with benzodiazepines/Z-drugs and no/minimal opioids as Group 3, and finally both benzodiazepines/Z-drugs and opioids as Group 4. Hazard ratios were calculated with the no-drug group as a reference, using Cox proportional hazards regression model adjusted for age, sex, number of chronic conditions and cancer patients excluded (n = 87,314).

**Results:** Hazard ratios for mortality increased both in Group 3 where it was 2.66 (95% CI 2.25–3.09) and in Group 4 where it was 5.12 (95% CI 4.25–6.17), with increased dose and higher number of chronic conditions. In Group 4 an opioid dose-dependent increase in mortality among persons using >1000 DDDs benzodiazepines/Z-drugs was observed when those on less than ≤300 DDDs of opioids with HR 4.94 (95% CI 3.54–6.88) were compared to those on >300 DDDs with HR 7.61/95% CI 6.08–9.55). This increase in mortality was not observed among patients on <1000 DDDs of benzodiazepines/Z-drugs.

**Conclusion:** The study supports evidence suggesting that mortality increases in a dose-dependent manner in patients co-prescribed benzodiazepines/Z-drugs and weak opioids (codeine, tramadol). An association between the number of chronic conditions and a rise in mortality was found. Long-term use of these drugs should preferably be avoided. Non-pharmacological therapy should be seriously considered instead of long-term use of benzodiazepines/Z-drugs, and deprescribing implemented for chronic users of these drugs when possible.

## Introduction

More than 10 years ago, the increase in the use of opioids in the United States was described as an epidemic (Okie, 2010) followed in recent years by several papers reporting on association between the use of these drugs and mortality, not least from the United States (Xu et al., 2020; Ray et al., 2021; Mooney et al., 2022). Although the increase in the prescribing of opioid analgesics in the US seems to be a result of an increasingly common treatment for pain (Park et al., 2015) these drugs are rarely prescribed alone indicating the complexity of the reasons for feeling pain. Thus, benzodiazepine receptor modulators (BZRM), i.e., benzodiazepines and Z-drugs are commonly prescribed concurrently for patients receiving opioid analgesics. Consequently, BZRMs could play a role in increased mortality.

A relationship has been found between overdose and concurrent use of opioids and benzodiazepines (Sun et al., 2017). Studies in general healthcare settings have shown increased risk for adverse events, emergency department visits, hospitalisation and mortality with chronic and high-dose use of opioids and concomitant use of benzodiazepines (Burgstaller et al., 2020; Häuser et al., 2020; Mooney et al., 2022).

There has been a trend in developed countries towards increased use of opioids and a shift towards stronger opioids like oxycodone (Zin et al., 2014). At the same time diminished prescribing of weak opioids has been observed (Fischer et al., 2018; Chenaf et al., 2019; Donovan et al., 2020). In spite of that, weak opioids are still the most prescribed opioids in many countries (Curtis et al., 2019; Nissen et al., 2019; Hurtardo et al., 2020; Olafsdottir et al., 2021). Thus, an escalation in prescriptions of strong opioids in primary care can be described as a matter of international concern, although in many countries using far less than the US or Canada, the term epidemic might be perceived as too strong a designation (Häuser et al., 2017). The opioid crisis in the US stands out in magnitude.

On an international level concurrent prescribing of opioids and benzodiazepines/Z-drugs is quite common and has been increasing (Larochelle et al., 2015; Torance et al., 2018). A number of papers have been published over the years concerning the association between the use of BZRMs and mortality without arriving at an agreement on causality, or up to which point enough confounding factors have been adjusted for. In a previous study we found an association between long-term use of hypnotics/anxiolytics and a dose-dependent increase in mortality (Linnet et al., 2019). The acute toxicity of both benzodiazepines and Z-drugs is low, but may be additive in various circumstances, for instance in individuals co-prescribed other psychotropic drugs (Chen et al., 2021; Kuo et al., 2022). High dosage and long-term use may increase the untoward effects. Medicalisation of sleeplessness may lead to unnecessary prescriptions of these drugs, thus increasing risks (Moloney et al., 2011; Moloney et al., 2019). The focus concerning all-cause mortality associated with opioid and benzodiazepine prescription has mostly been on strong opioids (Park et al., 2015; Sun et al., 2017). Nevertheless, some papers have been published concerning all-cause mortality where weak opioids have been co-prescribed with benzodiazepines (Jeong et al., 2019; Xie et al., 2021). A Canadian study revealed that adding benzodiazepines to any opioid molecule increased the risk of hospitalisation, emergency department visits or mortality (Sharma et al., 2020).

Thus, studies focusing on co-prescribing of weak opioids and BZRMs are scarce, not least regarding dose-dependent increase in mortality in long-term users of weak opioids and BZRMs, and the relative mortality of tramadol vs. codeine.

The aim of this study was therefore to analyse the association between on one side the long-term use (3 years) of BZRMs and/or mainly weak opioid analgesics, established during a 4-year exposure window, and on the other side the extent of long-term conditions and mortality in a cohort of primary care patients in the Reykjavik metropolitan area. Lastly, to find if all-cause mortality increased in a dose-dependent manner during a subsequent 5-year follow-up, and whether the mortality was associated with prescribing of different types of opioids.

## Materials and methods

### Design and setting

The 19 primary healthcare centres in the Reykjavik area with a population of just over 200,000, are staffed by general practitioners (GPs), midwives, nurses and other personnel. Information on age and sex of all patients who had contacted the primary healthcare centres during a 4-year period from 1 January 2009 to 31 December 2012 was retrieved from the collective medical records database of the primary healthcare centres, as well as the diagnosis (International Statistical Classification of Diseases and Related Health Problems 10th Version; ICD-10) for chronic conditions as described in two previous papers (Linnet et al., 2016; Linnet et al., 2019), shown in Table 1. The data in the medical records database is stored under a unique personal identifier (ID) allocated to every inhabitant.

**TABLE 1:** Chronic medical diseases/conditions according to ICD-10 considered relevant in this study.

Disease	ICD-10 code
Tuberculosis	A15–A19
Herpes zoster	B02
Human immunodeficiency virus	B20–B24
Cancer	C00–C97
Thyroidal diseases	E00–E07
Diabetes	E10–E14
Metabolic diseases	E65–E68
Hyperlipidaemia	E78
Mental health problems	F00–F99
Epilepsy	G40
Cardiovascular disease	I00–I09, I16–I99
Hypertension	I10–I15
Chronic obstructive pulmonary disease	J44
Asthma	J45–J46
Bronchiectasis	J47
Gastro-oesophageal reflux	K21
Psoriasis	L40
Rheumatoid arthritis	M05–M14
Osteoarthritis	M15–M19
Ankylosing spondylitis	M45
Chronic back pain	M53–M54
Fibromyalgia/myalgia	M79
Osteoporosis	M80–M82
Other chronic musculoskeletal problems	M00–M03, M20–M43, M46–M51, M60–M77, M83–M99
Renal disease	N18–N19

### Exposure

The extracted data from the medical records were linked through the patients’ IDs with data on redeemed prescriptions for benzodiazepines, Z-drugs and opioid analgesics in the Icelandic Medicines Registry (IMR) in the years 2008–2012. All redeemed prescriptions for benzodiazepines and Z-drugs with the Anatomical Therapeutic Chemical (ATC) codes N05BA (benzodiazepine anxiolytics), N05CD (benzodiazepine hypnotics-sedatives) and N05CF (benzodiazepine related hypnotics-sedatives) plus analgesic opioids with the ATC code N02A were retrieved. ICD-10 diagnoses 2009–2012 were retrieved from the medical records database of the Primary Healthcare of the Capital Area. Information on mortality in this cohort was extracted by linking the IDs with relevant data in the National Cause of Death Registry. All this data was collected in a separate study database. When all relevant data had been linked through the subjects’ IDs it was encrypted so the personal identity of the patients would not be revealed during the processing of the data set. The researchers did not at any time have any access to the personal identity of the participants, neither during the acquisition of the data nor during the anonymisation process performed by the Directorate of Health. Participants contacted the primary healthcare in the Reykjavik metropolitan area during 2009, and the follow-up started 3 years after the first prescription or the first visit. Persons who redeemed prescriptions for the above-mentioned drugs for 3 consecutive years were included and defined as consistent long-term users in our study. The cohort was divided into four groups as regards the period 1 January 2009 to 31 December 2012: Group 1 who neither used benzodiazepines, Z-drugs nor opioids during these years, Group 2 who continuously used opioids over a 3-year period with low or minimal intermittent use of benzodiazepines and/or Z-drugs. Group 3 comprised individuals with a continuous 3-year use of benzodiazepines and/or Z-drugs, but low or minimal use of opioids. Group 4 included persons with a continuous 3-year co-prescribing of opioids and benzodiazepines and/or Z-drugs during this period. The baseline characteristic and the main chronic conditions of the four groups are shown in Table 2.

**TABLE 2:** Comparison of the main baseline characteristics and chronic diseases in the four study groups. In general, the IMR shows that women are prescribed around 80% more of Z-drugs and approximately 110% more of benzodiazepines than men.

	Group 1^a^	Group 2 opioids	Group 3 BZRMs	Group 4 both
	No drugs			
Baseline characteristics				
Total number of participants	n = 78,248	n = 304	n = 7,881	n = 2,399
Men, n (%)	40,461 (52)	89 (29)	2,754 (35)	706 (29)
Women, n (%)	37,787 (48)	215 (71)	5,127 (65)	1,693 (71)
Average age (years)	33.5	43.8	53	53.4
SD of average age (years)	15.6	13.2	11.3	11.3
Chronic conditions				
Average number of diagnoses (n)	1.3	3.4	3.4	4.7
Average number of pain-related diagnoses (n)	0.7	1.7	1.4	2
Patients with no mental diagnoses, n (%)	66,733 (85)	129 (42)	2,009 (25)	537 (22)
Patients with mental diagnoses, n (%)	11,415 (15)	175 (58)	5,872 (75)	1,862 (78)
Number of chronic conditions				
Patients with 0–1 diagnosis, n (%)	49,808 (64)	65 (21)	1,783 (23)	392 (16)
Patients with 2–3 diagnoses, n (%)	20,765 (27)	100 (33)	2,437 (31)	403 (17)
Patients with 4–5 diagnoses, n (%)	6,087 (8)	89 (29)	2,218 (28)	648 (27)
Patients with 6–7 diagnoses, n (%)	1,297 (0.6)	32 (11)	1,051 (13)	543 (23)
Patients with 8–15 diagnoses, n (%)	291 (0.4)	18 (6)	392 (5)	413 (17)

a **Group 1:** No use of Benzos/Z-drugs nor opioids 2009–2012. **Group 2:** Long-term users of opioids only. **Group 3:** Only Benzos/Z-drugs for three consecutive years over the same period. **Group 4:** Long-term users of both Benzos/Z-drugs and opioids.

To find out whether the participants had a previous history of drug use, i.e., before the start of the research period, the prescribing of benzodiazepines, Z-drugs and opioids in 2008 was examined. Prior use of these drugs was divided into four categories, 1) low or minimal use of benzodiazepines, Z-drugs and opioids, 2) prior use of either benzodiazepines or Z-drugs only, 3) prior use of opioids only, and 4) prior use of benzodiazepines and/or Z-drugs as well as opioids. Low or minimal use was defined as ≤30 DDDs of each particular drug class, for prior history of only benzodiazepines and/or Z-drugs their use had to be >30 DDDs and use of opioids ≤30 DDDs. For prior history of opioids only their use had to be >30 DDDs and use of benzodiazepines and/or Z-drugs ≤30 DDDs. Finally, for being considered both benzodiazepine/Z-drug and opioid user, the redeemed benzodiazepines and/or Z-drugs had to be >30 DDDs and similarly prescribing of opioids >30 DDDs.

### Covariates

In this study we use previously described conditions as the frame of reference concerning chronic conditions, although we are not focusing specifically on multimorbidity (presence of two or more long-term health conditions in the same person) as such in this paper compared to the two preceding papers (Linnet et al., 2016; Linnet et al., 2019). In the three Cox proportional regression analyses, adjustment was made for age and sex in model 1, age, sex and number of chronic conditions in model 2. In model 3 all participants with confirmed cancer diagnoses were excluded and the same adjustments made as in model 2. Distribution of the total number in the four groups, age, sex and diagnoses can be seen in Table 2.

### Outcome

Information on mortality in the cohort was extracted by linking the IDs with relevant data in the National Cause of Death Registry. When all relevant data for the study database had been linked through the subjects’ IDs it was encrypted so the personal identity of the patients would not be revealed during the processing of the data set which was collected in a separate study database.

### Subject groups and statistical analysis

**FIGURE 1:**
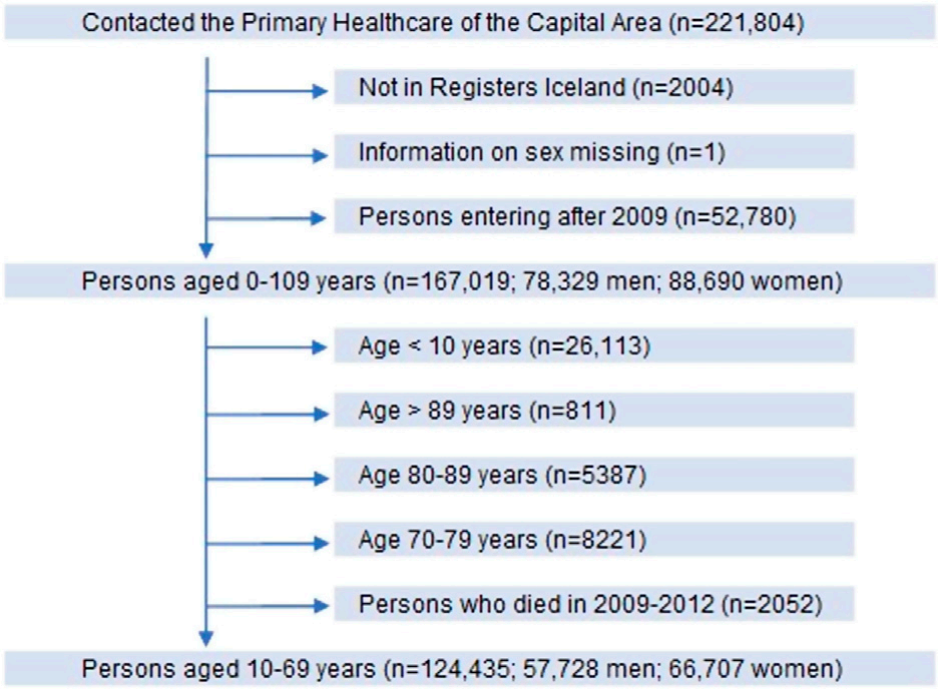
Flow chart of participants in the longitudinal cohort study in primary care in Iceland. Included participants shown by vertical arrows and those excluded by horizontal arrows.

Participants in the study were 10–69 years old legal residents with domicile in Iceland, and thus health-insured, who attended primary healthcare centres in the Reykjavik metropolitan area during the period 2009–2012. After exclusion of those who did not meet the inclusion criteria, we ended up with 124,435 persons, 46% men and 54% women as shown by the flow chart in Figure 1.

**FIGURE 2:**
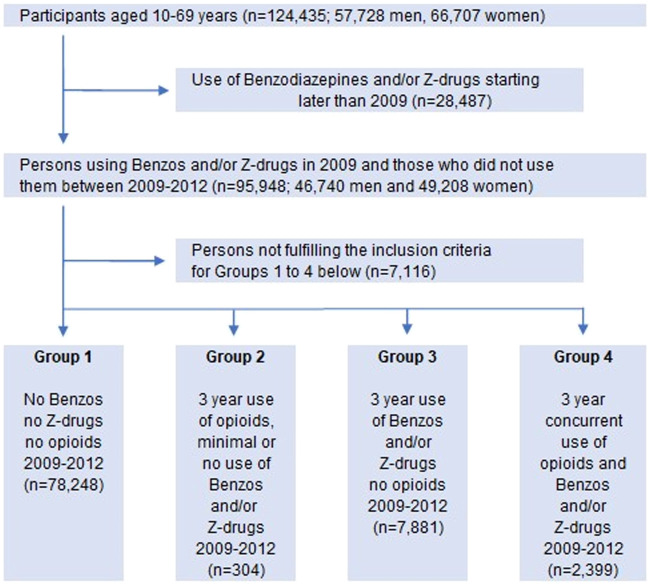
Flow chart of participants divided into four groups according to different prescribing/(use) of benzodiazepines, Z-drugs and opioids. Vertical arrows: included; horizontal arrows: excluded.

The participants were divided into four groups according to their use of benzodiazepines, Z-drugs and opioids as shown in Figure 2. To determine whether there was an association between long-term prescribing of these drugs on one side and chronic conditions and excess mortality on the other side, we followed the groups until the end of year 2018, setting Group 1 (no drug use) as a reference.

We also examined if increased morbidity and mortality was associated in a dose-dependent manner with long-term prescribing of benzodiazepines and/or Z-drugs and opioids, i.e., DDDs/person/3 years in Groups 3 and 4. The groups were divided according to the median long-term use of the drugs in each group. In Group 3 there were two subgroups, either those using benzodiazepines and/or Z-drugs ≤600 DDDs or those using >600 DDDs. Group 4 (benzodiazepines, Z-drugs and opioids) was divided into four subgroups with benzodiazepines/Z-drugs either ≤1000 or >1000 DDDs, and the opioid subgroups using either ≤300 or >300 DDDs.

Two sensitivity analyses were performed. Firstly, to find if the association between use of benzodiazepines, Z-drugs and opioids on one hand and morbidity and mortality on the other depended upon which kind of opioids patients in Group 4 used. This was a two-step procedure. In step a. all patients using strong opioids in years 2009–2012 regardless of doses or timespan were excluded, so only those using codeine or tramadol were left. In step b. Patients using tramadol were excluded, leaving codeine as the only opioid. Secondly, it was examined whether the association between use of benzodiazepines, Z-drugs and opioids versus morbidity and mortality would be sensitive to changes in the age criteria in the cohort. It was a two-step process: the first step included participants 10–59 years of age, and in the second step they were aged 30–59 years.

Statistical analyses were conducted with the software RStudio version 1.2.5033. Right censoring occurred when follow up stopped at end-of-study 31 December 2018. Statistical significance was set at 0.05.

## Results

At study entry the number of participants was 124,435; 57,728 men and 66,707 women, ranging between 10 and 69 years of age (Figure 1). When those who did not meet the inclusion criteria were excluded, the remaining 88,832 persons (44,010 men, 44,822 women) in the study population were divided into four groups according to their use of benzodiazepines, Z-drugs and opioids (Table 2; Figure 2).

The relationship between long-term use of benzodiazepines, Z-drugs and opioids on the one hand and morbidity and mortality on the other was estimated with Cox models 1–3. The follow-up was 81–2,556 days (up to 7 years), average time 2,419 days (ca 6 years and 8 months) and the median 2,471 (ca 6 years and 9 months). In Cox model 3, 1,214 cancer patients were excluded from the cohort. All the Cox models showed that at all times long-term use of benzodiazepines, Z-drugs and opioids increased the mortality risk compared to no use of these drugs.

Model 3 (no cancer patients) showed almost a threefold increase in the risk for Group 3 (benzos/Z-drugs), while the risk in Group 4 (benzos/Z-drugs/opioids) increased fivefold compared to non-users. The hazard ratio for Group 4 is up to twice the hazard ratio for Group 3 and the confidence interval in Group 4 is more than twice as wide, indicating greater inaccuracy (Table 3). Group 2, long-term users of opioids only, was omitted from the calculations as the patients in this group were too few (304) with less than 10 deaths, so statistical calculations for this group were not meaningful (Figure 2).

**TABLE 3:** The relationship between long-term use of hypnotics-sedatives, hypnotics-sedatives/opioids, morbidity and mortality. Cox proportional hazards regression is used for models 1–3.

	No. of persons	No. of deaths	HR	95% CI
Model 1—adjusted for age and sex				
All Groups (total)	88,528	1,186		
Group 1	78,248	524	1.00	Ref
Group 3	7,881	405	2.92	2.54–3.40
Group 4	2,399	257	6.27	5.35–7.40
Model 2—adjusted for age, sex and number of chronic conditions				
All Groups (total)	88,528	1,186		
Group 1	78,248	524	1.00	Ref
Group 3	7,881	405	2.73	2.37–3.15
Group 4	2,399	257	5.36	4.51–6.37
Model 3—cancer patients excluded, adjusted for age, sex and number of chronic conditions				
All Groups (total)	87,314	1,039		
Group 1	77,593	488	1.00	Ref
Group 3	7,507	343	2.66	2.28–3.09
Group 4	2,214	208	5.12	4.25–6.17

**Group 1:** No use of Benzos/Z-drugs nor opioids 2009–2012. **Group 3:** Only Benzos/Z-drugs for three consecutive years over the same period. **Group 4:** Long-term users of both Benzos/Z-drugs and opioids.

The long-term users of benzodiazepines, Z-drugs and opioids in Groups 3 and 4 were subdivided into different dose categories. In Group 3 there were two subgroups, either those using benzodiazepines and/or Z-drugs ≤600 DDDs or those using >600 DDDs. Group 4 (benzodiazepines, Z-drugs and opioids) was divided into four subgroups with benzodiazepines/Z-drugs either ≤1000 or >1000 DDDs, and the dose of opioids either ≤300 or >300 DDDs (Table 4). This was done in order to estimate the relationship between long-term use of these drugs and the influence of dosage on the mortality.

**TABLE 4:** Dose-dependent relationship between long-term use of benzodiazepines/Z-drugs, opioids, morbidity and mortality. Cox proportional hazards regression is used for models 1–3, Groups 3 and 4.

Model 1—adjusted for age and sex					Model 2—adjusted for age, sex and number of chronic conditions		Model 3—cancer patients excluded, adjusted for age, sex and number of chronic conditions			
	No. of persons	No. of deaths	HR	95% CI	HR	95% CI	No. of persons	No. of deaths	HR	95% CI
All Groups (total)	88,528	1,186					87,314	1,039		
Group 1	78,248	524	1.00	Ref	1.00	Ref	77,593	488	1.00	Ref
Group 3 - DDD ≤600 BZD/Z-drugs	4,106	137	2.05	1.69–2.50	1.96	1.61–2.39	3,934	112	1.82	1.47–2.26
Group 3 – DDD	3,775	268	3.73	3.19–4.40	3.47	2.95–4.07	3,573	231	3.46	2.92–4.10
>600 BZD/Z-drugs										
Group 4 – DDD	650	47	4.47	3.30–6.10	3.97	2.91–5.40	608	33	3.19	2.22–4.58
≤1000 BZD/Z-drugs										
≤300 Opioids										
Group 4DDD	345	26	4.25	2.86–6.30	3.71	2.48–5.54	312	18	3.08	1.91–4.98
≤1000 BZD/Z-drugs										
>300 Opioids										
Group 4—DDD	480	50	5.82	4.33–7.80	5.11	3.77–6.92	442	41	4.94	3.54–6.88
>1000 BZD/Z-drugs										
≤300 Opioids										
Group 4—DDD	924	134	8.52	7.00–10.40	7.32	5.93–9.03	852	116	7.61	6.08–9.52
>1000 BZD/Z-drugs										
>300 Opioids										

Group 1: Individuals who neither used benzodiazepines, Z-drugs nor opioids during 2009–2012. Group 3: Those who used only benzodiazepines and/or Z-drugs for three consecutive years over the same period. Group 4: Long-term users of both benzodiazepines and/or Z-drugs and opioids.

The three Cox regression models show an increased mortality risk for higher doses of benzodiazepine/Z-drugs only (Group 3). In model 3 the HR increased almost twofold from 1.82 (95% CI 1.47–2.26) in the <600 DDDs/3 years subgroup to 3.46 (95% CI 2.92–4.10) in the subgroup using >600 DDDs/3 years. A dose-dependent increase in mortality risk was also observed in the benzodiazepine/Z-drug/opioid group (Group 4). When the dose of benzodiazepine/Z-drug was ≤1000 DDDs/3 years there was only a minimal difference in HR when the opioid dose changed from ≤300 DDDs/3 years to >300 DDDs/3 years. With Benzos/Z-drugs >1000 DDDs/3 years an increase in the dose of opioids from ≤300 DDDs/3 years to >300 DDDs/3 years increased the HR from 4.94 (95% CI 3.54–6.88) to 7.61 (95% CI 6.08–9.52), i.e., there was up to almost a twofold increase in mortality. Table 4 shows that the 95% CI was considerably wider in Group 4 (Benzo/Z-drug-opioids) than in Group 3 (Benzo/Z-drugs only), pointing to a greater uncertainty in mortality risk assessment.

Two sensitivity analyses were performed. The first analysis was a two-step process for examining the different weak opioids and their relationship with mortality. In this case participants using strong opioids had been excluded from Group 4. Thus, in step one both tramadol and codeine users were included. In step two the tramadol users were excluded so only the codeine users were left. Excluding the strong opioids users resulted in ca 20% reduction in the number of persons in the cohort, and a further 19% reduction when the cancer patients were excluded. The HR in Cox model 3 lowered from 5.12 (95% CI 4.25–6.17) to 4.52 (95% CI 3.69–5.55), i.e., the mortality risk diminished about 12% at each point of time. When tramadol users were excluded as well, the number of persons was lowered about 62% and with the exclusion of cancer patients the reduction was 61%. The HR in Cox model 3 was reduced by 36% from 5.12 (95% CI 4.25–6.17) to 3.30 (95% CI 2.48–4.42) when there were only codeine users left in the cohort. In short, excluding users of strong opioids decreased the mortality risk by 0.6 (HR), and when tramadol users were excluded as well the risk was further reduced by 1.22 (HR). Nevertheless, long-term use of codeine was associated with increased mortality in the group of patients who were co-prescribed BZRMs and codeine, although to a lesser degree than in the combined group of strong and weak opioids. It may draw some attention that in this group of patients, strong opioids increase mortality only by 12%, which probably may be explained by the relatively low number (n = 489) of the long-term users of benzodiazepine modulators and opioids (n = 2,399) who were prescribed strong opioids for one, two or 3 years.

The second sensitivity analysis was also a two-step process where we looked at the relationship between the use of benzodiazepines/Z-drugs and opioids on the one hand, and morbidity and mortality on the other hand to find if it was sensitive to changes in the age interval. In the first step the age criterium was 10–59 years of age, i.e., participants aged 60–69 years were excluded. In the second step participants aged 10–29 years were also excluded so the age interval in that step was 30–59 years. In the first step participants in Group 1 became 7% fewer, in Group 3 34%, and 36% fewer in Group 4 (both participants with and without cancer diagnoses). When Table 5 is compared with Table 3, it can be seen that changes in age criteria increase the HRs in all the groups, and that it increases considerably more in Group 4 than in the other groups. In Group 3 it increases from 2.66 (95% CI 2.28–3.09) to 3.9 (95% CI 3.09–4.87), and in Group 4 it increases from 5.12 (95% CI 4.25–6.17) up to 8.2 (95% CI 6.24–10.90).

**TABLE 5:** Sensitivity analyses. Cox proportional hazards regression is used for models 1–3. Category I. Different weak opioids: Group 4, strong opioids excluded: A. Weak opioids: tramadol/codeine. B. Codeine only. Category II. Different age intervals. Groups 3 and 4: A. Participants aged 10–59 years and B. 30–59 years respectively.

Model 1					Model 2		Model 3			
	No. of persons	No. of deaths	HR	95%CI	HR	95%CI	No. of persons	No. of deaths	HR	95%CI
Category I—different weak opioids										
*A: Weak opioids: tramadol/codeine*										
	(80,158)	(709)					(79,375)	(643)		
Group 1	78,248	524	1.00	Ref	1.00	Ref	77,593	488	1.00	Ref
Group 4	1,910	185	5.44	4.55–6.5	4.62	3.82–5.58	1,782	155	4.52	3.69–5.55
*B: Codeine only*										
	(79,158)	(593)					(78,446)	(545)		
Group 1	78,248	524	1.00	Ref	1.00	Ref	77,593	488	1.00	Ref
Group 4	910	69	4.10	3.20–5.35	3.45	2.64–4.50	853	57	3.30	2.48–4.42
Category II—different age intervals										
*A. Aged 10–59* *years*										
	(79,605)	(548)					(78,959)	(491)		
Group 1	72,855	272	1.00	Ref	1.00	Ref	72,456	260	1.00	Ref
Group 3	5,221	162	4.32	3.50–5.33	3.99	3.22–4.96	5,073	142	3.90	3.09–4.87
Group 4	1,529	114	11.19	8.86–14.12	9.19	7.12–11.87	1,430	89	8.20	6.24–10.90
*B. Aged 30–59* *years*										
	(43,230)	(484)					(42,630)	(427)		
Group 1	36,918	222	1.00	Ref	1.00	Ref	36,564	210	1.00	Ref
Group 3	4,866	153	3.84	3.09–4.76	3.58	2.87–4.48	4,719	133	3.44	2.72–4.34
Group 4	1,446	109	10.09	7.94–12.81	8.40	6.46–10.91	1,347	84	7.42	5.57–9.88

Long-term use of hypnotics-sedatives and opioids (Group 4) where those using strong opioids have been excluded, leaving in the first place only participants using weak opioids (A), and secondly after excluding tramadol users only codeine users were left (B). Group 3: Long-term use of benzodiazepines/Z-drugs. Model 1—adjusted for age and sex. Model 2 same as 1 + chronic condition. Model 3 same as 2 but cancer patients excluded.

In the second step (30–59) participants in Group 1 both with and without cancer diagnoses became 53% fewer, in Group 3 38% fewer and in Group 4 they became 40% fewer. This change in the age interval influences the association between the long-term use of the drugs and mortality and morbidity in all the Cox models, but slightly less when compared to the 10–69 years age interval. In Model 3 it rises from HR = 2.66 (95% CI 2.28–3.09) to HR = 3.44 (95% CI 2.72–4.34) and in Group 4 the corresponding values are from 5.12 (95% CI 4.25–6.17) to 7.42 (95% CI 5.57–9.88). Thus, the HRs increased in all age intervals in both Group 3 and Group 4 when compared to Group 1, and most in the 10–59 years age interval in Group 4.

When looking at prior history of the use of benzodiazepines/Z-drugs and opioids in 2008 (Table 6) there was minimal use of these drugs in Group 1 (Ref. group). In the opioid-users’ group (Group 2) prior history revealed some use of opioids, but very low use of benzodiazepines/Z-drugs. In Groups 3 and 4 the use of drugs from both categories was quite common in 2008 while the use of opioids was quite modest, especially in Group 3. This shows that there is at least a 4-year continuous use in these groups, and in accordance with previously reported research this suggests that earlier use of benzodiazepines/Z-drugs may predict repeated use of opioids (Skurtveit et al., 2010).

**TABLE 6:** Prior history of benzodiazepine/Z-drug and opioid use in the four study groups in 2008.

*Prior history of use*	Group 1	Group 2	Group 3	Group 4
None/minimal, n (%)	77,019 (98.4)	191 (62.8)	2,095 (26.6)	194 (8.1)
Opioids, n (%)	932 (1.2)	74 (24.3)	46 (0.6)	157 (6.5)
BZD/Z-drugs, n (%)	263 (0.3)	21 (6.9)	5,496 (69.7)	544 (22.7)
BZD/Z-drugs + Opioids, n (%)	34 (0.04)	18 (5.9)	244 (3.1)	1,504 (62.7)
Total, n (%)	78,248 (100)	304 (100)	7,881 (100)	2,399 (100)

Group 1: Individuals who neither used benzodiazepines/Z-drugs nor opioids during 2009–2012.

Group 2: Long-term users of opioids with continuous use (3 years) in the period 2009–2012.

Group 3: Long-term users of benzos/Z-drugs with continuous 3 years use in the period 2009–2012.

Group 4: Long-term users of both benzos/Z-drugs and opioids with a continuous use (3 years) of drugs in both categories (N02A and N05A) during the period 2009–2012.

## Discussion

### Principal findings

Participants on long-term (3 years) opioid and BZD/Z drug treatment were consistently at increased risk of all-cause mortality. There was an opioid dose-dependent increase in mortality among patients on long-term concomitant treatment with opioids and high dose (>1000 DDDs/3 years) BZD/Z-drug treatment when persons on less than ≤300 DDDs of opioids were compared to those on >300 DDDs. This increase in mortality was not observed among subjects on lower dose (<1000 DDDs) of BZD/Z-drugs. When all those on long term opioid and BZD/Z-drug treatment were analysed as one group, the risk of mortality decreased by 12% when persons on strong opioids were excluded. When subjects on tramadol were excluded leaving only codeine the risk was reduced further by 27% indicating that the risk associated with tramadol use may be closer to that of the strong opioids than to codeine.

### Strengths

This is a comprehensive study covering approximately two-thirds of the total population where all contacts with all the primary healthcare centres in the area are registered in the same medical records database. The distribution according to age and sex is the same as for the population at large so the cohort is considered to be representative. By linking the IDs to the data on redeemed prescriptions in the Icelandic Medicines Registry during the study period we were able to cover all prescriptions for the relevant drugs issued by the GPs, claimed by patients in the Reykjavik metropolitan area.

### Limitations

The same list of chronic conditions was used as in our previous research papers (Linnet et al., 2016; Linnet et al., 2019) where the focus was on multimorbidity regarding the diseases. It may be argued that more diagnoses should have been added to this collection. The burden of disease can vary depending on the severity and aetiology of the disease, age of the patient, and so on, making the division into groups according to the number of chronic conditions somewhat ambiguous. This can influence the outcome, including mortality. Potential confounders that we lack information on, include use of other respiratory suppressant drugs or sedatives and other fall-risk increasing drugs, alcohol consumption, smoking, social status, education and income (social variables are not registered in medical records). Moreover, confounding by indication cannot be excluded. Considering confounders not registered in the current study and residual confounding, we might expect the HRs to attenuate to some degree. On the other hand, we assume non-adherence to treatment to be present similar to other pharmacoepidemiological studies. If non-adherence was not present, the HRs observed in the current study would be somewhat higher. We assume patients took the prescribed medications they redeemed. Our data did not include individual prescriptions, but the sum of drug use in DDDs during each 365 days’ period from the first BZRM prescription in the exposure window (2009–2012). In order to estimate the prevalence of concurrent use of benzodiazepine/Z-drugs (N05C hypnotics-sedatives) and opioids (N02A), all individuals in Iceland 18 years of age and older who received one or more prescriptions of BZRMs and/or opioids in 2009 were counted, along with those receiving one or more prescriptions within a 30-day period. The number of co-prescriptions during 2009 were 13,950 and the number of persons getting co-prescriptions 30 days or less apart were 10,916 (78.3%). The corresponding values for anxiolytic benzodiazepines (N05B) and opioids were 9,012 within 1 year and 6,168 within 30 days (68.4%). Our data include only prescriptions claimed at pharmacies, not prescriptions issued for the treatment of patients in hospitals.

### Diseases

In our previous studies (Linnet et al., 2016; Linnet et al., 2019) we found that the likelihood of being prescribed Benzos/Z-drugs increased considerably with multimorbidity. When patients were grouped by number of chronic conditions the risk of mortality increased with increasing number of chronic conditions. This study shows an additive increase in mortality for patients taking both BZRMs and opioids with a further increase, the higher the number of chronic conditions is.

### Drug dose, drug combinations and morbidity/mortality

We defined long-term use of both BZRMs and opioids as a 3-year consistent use relatively evenly spread over a 3-year period. Regulations only permit a month’s supply of these drugs to be dispensed each time a patient presents in the pharmacy claiming a prescription. Thus, consistent users will get them on a regular basis evenly spread throughout the year. We were particularly looking at the co-prescribing of BZRMs and opioids, which is much more common than prescribing of opioids alone. In Iceland almost 66% of the opioids prescribed in 2020 was codeine in combinations with paracetamol, 21% was tramadol, 5.1% oxycodone and 4.1% morphine (Directorate of Health, 2021). Thus, around 87% of the opioids are weak opioids. Our cohort therefore provides a good opportunity to examine the association of co-prescribing of weak opioids and BZRMs with mortality risk and increased morbidity. In this study as the dose of BZRMs increased so did the mortality, consistent with the results in our previous study (Linnet et al., 2019) and when the opioids were added, the mortality risk increased even more.

### Pharmacological considerations

The pharmacological properties of both BZRMs and opioids could play a role in mortality. Although respiratory compromise is uncommon in isolated ingestions of BZRMs we are dealing with consistent long-term use where a number of other drugs or substances can come into consideration where the respiratory suppressant effects could be augmented. This could be relevant in the case of opioids where respiratory depression is a well-known side effect often with serious consequences. The use of psychotropic drugs can lead to increased risk of falls and fractures (Waade et al., 2017; Ohara et al., 2021) and as a consequence increased mortality in the elderly (Shaver et al., 2021). Increased suicide risk has been reported (Cato et al., 2019) as well as risk of traffic accidents (Booth et al., 2016). Pulmonary diseases like COPD and pneumonia may play a role (Obiora et al., 2013; Baillargeon et al., 2019) as well as increased disease burden in general. There is a number of individual factors such as dosage, tolerance, age, weight, social factors and even genetic variation that can further complicate the picture. Weighing risks against benefits beyond the few weeks use of BZRMs recommended in clinical guidelines, we have not been able to find papers documenting evidence of beneficial effects of long-term use of these drugs. On the contrary, as referred above numerous papers have shown an association between their use and various untoward effects. In a position paper of the American Academy of Neurology (Franklin, 2014) it is stated that although there is evidence for significant pain relief in the short term in patients treated with opioids, there is no substantial evidence for maintenance of pain relief in the long run. Opioid-induced hyperalgesia could also pose a significant clinical challenge in long-term treatment with opioids (Tompkins and Campbell, 2011). In addition to the above-mentioned risks, addiction is a well-known problem of both opioids and BZRMs.

### Implications

Non-pharmacological approaches such as cognitive behavioural therapy should be advised instead of long-term use of BZRMs, and it should be carefully considered whether a possible benefit of paracetamol-codeine combinations for pain management outweigh the benefit of using paracetamol alone, considering the comparatively small increase in analgesia of the combination product and the increased incidence of side effects occurring with repeated use (de Craen et al., 1996). Long-term co-prescribing of BZRMs and opioids, weak or strong, should preferably be avoided. Long-term use of weak opioids may have been considered relatively harmless in the clinical setting as shown by over-the-counter status in many countries, but this view needs to be reconsidered by clinicians as well as by the authorities. Deprescribing inappropriate medicines where patients have developed addiction can be challenging, but should be addressed in a stepped approach with a patient-centred focus included in the framework (Reeve, 2020).

## Summary and conclusion

There was a dose-dependent increase in mortality associated with long-term prescribing of BZRMs which was augmented with long-term co-prescribing of opioids, both tramadol and codeine, although to a lesser degree in the case of codeine. There was also an association with the number of chronic conditions, the higher their number, the higher was the mortality. Our findings support non-pharmacological treatment as first-line treatment for insomnia. When pharmacological treatment is necessary, long-term BZRMs alone or in combination with opioids should be avoided to reduce the risk of increased mortality and other untoward effects. Recommendations of clinical guidelines should be followed.

## Data Availability

The datasets presented in this article are not readily available because: The data is retrieved from a medical records database in the Primary Healthcare of the Capital Area, the Icelandic Medicines Registry and the National Cause of Death Registry of the Directorate of Health, and is not publicly available. The encrypted data is kept at the Directorate of Health and can be made available on a reasonable request if permitted by the above-mentioned health authorities. Requests to access the datasets should be directed to www.landlaeknir.is and visindanefnd@heilsugaeslan.is.
